# Atypical facial pain in multiple sclerosis caused by spinal cord seizures: a case report and review of the literature

**DOI:** 10.1186/s13256-016-0891-x

**Published:** 2016-04-20

**Authors:** Kunal Gupta, Kim J. Burchiel

**Affiliations:** Department of Neurological Surgery CR-137, Oregon Health and Sciences University, 3181 SW Sam Jackson Park Road, Portland, OR 97239 USA

**Keywords:** Multiple sclerosis, Trigeminal neuralgia, Spinal cord seizures, Pain

## Abstract

**Background:**

Pain is a very commonly reported symptom and often drives patients to seek medical attention; however, it can prove a very difficult diagnostic conundrum and even more challenging to treat effectively. Accurately determining the primary pain generator is key, as certain conditions have efficacious medical and surgical treatments. We present a rare case of a man with multiple sclerosis presenting with spinal cord seizures causing dermatomal pain. While pain has been reported in the context of motor symptoms attributed to spinal cord seizures in a small number of spinal cord conditions, this case represents the first report of pain exclusively associated with spinal cord demyelination in multiple sclerosis.

**Case presentation:**

We present the case of a 60-year-old Caucasian male patient with multiple sclerosis who reported a 5-year history of progressive pain in his left retroauricular region and superior left shoulder. He described this pain as sharp, episodic, and unrelenting and he was referred for consideration for surgical treatment of trigeminal neuralgia. He had no evidence of trigeminal nerve root pathology on magnetic resonance imaging, but did show dorsolateral spinal cord demyelination at the C3–4 level. His symptoms therefore represent an unusual presentation of spinal cord seizures.

**Conclusions:**

Spinal cord seizures are rarely reported in multiple sclerosis and typically present with focal motor seizures. These have been reported to present with cramping dysesthesia and pruritus, though rarely with primary pain. Knowledge of uncommon pain presentations is critical for the increasing number of primary care physicians caring for patients with such chronic neurological diseases as it will guide management and referral patterns. This knowledge is also important for the treating neurologists and neurosurgeons. Neurosurgical intervention for trigeminal neuralgia poses considerable surgical risk, and it should be avoided where possible. Identifying the primary pain generator is, therefore, critical for accurate diagnosis and management.

## Background

Pain in the setting of multiple sclerosis (MS) is increasingly recognized, and can be caused by a range of pain syndromes, including headache and facial pain, Lhermitte’s sign, and neuropathic body pain, as well as pain related to tonic spasms and spasticity [1]. One prominent and well-recognized cause of facial pain in MS is trigeminal neuralgia. Trigeminal neuralgia is frequently described by patients as a lancinating pain and/or burning dysesthesia and occurs in the facial trigeminal nerve dermatomes. While it is well recognized in the context of MS, trigeminal neuralgia occurs infrequently, with a prevalence of 1–3 % of patients with MS. A range of etiologies of trigeminal nerve dysfunction has been identified in the context of MS, including mass effect from tumors and neurovascular compression. However, a direct cause associated with MS is believed to be the demyelination of the trigeminal nerve root entry zone at the brainstem. The surgical management of trigeminal neuralgia in the setting of neurovascular compression is typically by open microvascular decompression; however, in the setting of MS, first-line management is often by percutaneous gangliolysis.

Our neurosurgery service received a referral for a 60-year-old man with MS for an unusual presentation of facial pain. He was referred with a diagnosis of trigeminal neuralgia and a request for surgical intervention. Upon evaluation, we noted that he had no evidence of trigeminal nerve root compression or demyelination on MRI, and clinically his pain was atypical for trigeminal neuralgia. We further reviewed an MRI of his cervical spine and noted dorsolateral demyelination at the clinically afflicted dermatome, leading to a diagnosis of spinal cord seizures, a rare and infrequently disorder associated with spinal cord demyelination in MS. Spinal cord seizures have also been reported in transverse myelopathy, traumatic spinal cord injury, and gliomas of the spinal cord [2–4]. The clinical manifestations reported are predominantly focal motor symptoms, including myoclonus and tonic spasm, occasionally accompanied by painful dysesthesia and pruritus [3]. Transient pruritus has been described in the setting of spinal cord glioma in a dermatomal pattern; this was responsive to gabapentin, leading the authors to hypothesize a neurological basis for the symptoms [4]. Prior case reports of patients with MS have documented spinal cord seizures with motor phenomena that afflict the myotomes corresponding with the region of the spinal cord affected by the demyelinated plaques [2, 3]. Primary pain syndromes caused by spinal cord demyelination are far less frequently reported [1, 5], and are thus they are often poorly recognized. We present this man’s case, discuss how these pain syndromes can be diagnostically differentiated, and evaluate the current medical and surgical treatment options.

## Case presentation

A 60-year-old Caucasian man with a diagnosis of relapsing-remitting MS was evaluated in our Neurosurgery outpatient clinic. He described his initial symptoms as beginning 7 years ago, with altered sensorium that occurred while driving. He developed mild left-fingertip numbness 2 years later. An MRI of his brain was performed at that time to aid in diagnosing his neurological symptoms. This demonstrated periventricular white matter lesions, and he was diagnosed with MS. He was initially treated with interferon-beta; however, recently he has been experiencing injection fatigue and stated that he is considering a transition to oral medications. He also described left-sided ear pain and shoulder dysesthesia, which began 5 years ago. He had tried multiple medications for this neuropathic pain, including gabapentin, pregabalin, and carbamazepine, with little clinical relief. Based on the described location of the pain, he was diagnosed with trigeminal neuralgia and referred to our Neurosurgery service for consideration for surgical treatment.

**Fig. 1:**
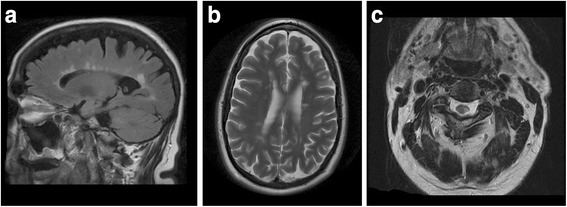
Magnetic resonance imaging of the brain. **a** A sagittal fluid attenuation inversion recovery sequence and **b** axial T2-weighted sequence demonstrate white matter lesions consistent with a diagnosis of multiple sclerosis. **c** A T2-weighted magnetic resonance image of his cervical spine demonstrates a lesion at the level of C3–4 affecting the left-sided dorsal horn and dorsolateral white matter tracts

On detailed questioning, our patient described episodic rapid-onset pain in his left shoulder that lasted 30 seconds and radiated to his ear and retroauricular region. These episodes did not have a trigger and had been progressively worsening. On a systematic review we noted that he described difficulty coordinating his left arm and leg over the past 5 years as well, corresponding to the duration of his pain. On examination he was alert and oriented, and recalled his history well. He showed no abnormal signs on a cranial nerve examination. Examination of his extremities demonstrated subtle left deltoid muscle wasting and weakness. His fine touch perception showed a left-sided patchy loss in his left C3–5 dermatomes. In his lower extremities he had a positive crossed-adductor response on his left leg, but no clonus was elicited and his reflexes were otherwise normal. An MRI of his brain and cervical spine demonstrated multiple demyelinated plaques in his corpus callosum, particularly prominent in his splenium, and a plaque on his left cervical spinal cord at the level of C3–4 in the dorsolateral column and dorsal horn (Fig. 1). He had no evidence of neurovascular compression or demyelination at his trigeminal nerve root entry zone.

## Discussion

We present the case of a patient with atypical facial pain in the setting of MS. He received a presumptive diagnosis of trigeminal neuralgia and was referred for surgical management. Upon review, we felt that his symptomatology was atypical for trigeminal neuralgia, with it predominantly affecting non-trigeminal dermatomes. A careful review of his MRI scan showed no evidence for trigeminal nerve pathology, and we identified an area of demyelination in the dorsolateral region of his cervical spinal cord in the region of his pain, which could have been localized to C3–4 dermatomes. We concluded that this region was the source of his pain and that his symptoms were a manifestation of spinal cord seizures. We therefore report a rare case of pain as a presentation of MS-associated spinal cord seizures.

Spinal cord seizures typically present with focal motor symptoms [3]; while burning dysesthesia has been described in the setting of tonic muscle spasms [2], episodic pain has not previously been reported as a primary presentation of spinal cord seizures. Our patient posed a diagnostic dilemma due to the clinical overlap in his symptoms with trigeminal neuralgia. We further discuss how these two syndromes can be diagnostically differentiated and the current medical and surgical treatment options. This case provides a critical opportunity to highlight patterns of symptomatology that can help distinguish between these two difficult-to-diagnose clinical entities, and direct investigation and treatment.

Trigeminal neuralgia is an uncommon occurrence in the setting of MS, affecting only 1–3 % of patients with MS. The mainstay of diagnosis and investigation for trigeminal neuralgia is by extensive clinical history, identifying the nature of the pain as well as localization to the trigeminal nerve distribution. MRI and MRA of the brain can identify compressive vascular structures, plaques, tumors, and other lesions affecting the trigeminal root entry zone.

Spinal cord seizures have been reported in MS, as well as in transverse myelopathy and traumatic spinal cord injury. These typically manifest as focal motor symptoms or myoclonus and tonic spasm, and they are occasionally accompanied by painful dysesthesia [2, 3]. Primary pain syndromes caused by spinal cord demyelination are rarely reported and poorly recognized; careful clinical examination and identifying spinal cord plaques on MRI can help guide diagnosis. Nerve conduction studies can also be helpful in excluding other conditions with similar presentations, such as radiculopathy attributable to nerve root compression. In our case, dermatomal pain outside the trigeminal dermatomes and the presence of a corresponding lesion in the dorsal horn of his cervical spinal cord led to the diagnosis of spinal cord seizures.

The substantia gelatinosa of the spinal cord and the spinal nucleus of the trigeminal nerve share both anatomical continuity and mechanisms in pain processing and the induction of neuropathic pain [6, 7]. Studies have demonstrated that transient receptor potential A1 and transient receptor potential vanilloid 1 are present in both locations and play a role in the communication of noxious stimuli to central pathways [8]. Furthermore, neuronal transduction of noxious stimuli is modulated by c-fos in both the trigeminal nerve and the substantia gelatinosa [9, 10]. It is therefore possible that the processes that underpin the development of neuropathic pain in both pathologies share mechanistic overlap.

Both spinal cord seizures and trigeminal neuralgia can be treated medically with anticonvulsant medications [11, 12]. While trigeminal neuralgia can be treated surgically by microvascular decompression/neurolysis or gangliolysis [13], proven surgical treatments for spinal cord seizures are currently lacking. Experimental procedures include vagal nerve stimulation and spinal cord stimulation [14, 15]. Therefore, accurate diagnosis of the primary pain generator is critical in guiding therapy.

## Conclusions

We have highlighted the diagnostic challenge posed by pain in patients with MS. We report an atypical presentation of pain caused by spinal cord seizures in the setting of MS, highlight the clinical distinction of spinal cord seizures from trigeminal neuralgia, and discuss the implications of accurate diagnosis for investigation and management.

## Consent

Written informed consent was obtained from the patient for publication of this case report and accompanying images. A copy of the written consent is available for review by the Editor-in-Chief of this journal.
